# A Potential Novel Treatment for Chronic Cough in Long COVID Patients: Clearance of Epipharyngeal Residual SARS-CoV-2 Spike RNA by Epipharyngeal Abrasive Therapy

**DOI:** 10.7759/cureus.33421

**Published:** 2023-01-05

**Authors:** Kensuke Nishi, Shohei Yoshimoto, Takayuki Tanaka, Shoichi Kimura, Yudai Shinchi, Takafumi Yamano

**Affiliations:** 1 Section of Otolaryngology, Department of Medicine, Fukuoka Dental College, Fukuoka, JPN; 2 Department of Otolaryngology, Faculty of Medicine, Fukuoka University, Fukuoka, JPN; 3 Otolaryngology, Nishi Otolaryngology Clinic, Fukuoka, JPN; 4 Section of Pathology, Department of Morphological Biology, Fukuoka Dental College, Fukuoka, JPN; 5 Oral Medicine Research Center, Fukuoka Dental College, Fukuoka, JPN; 6 Department of Pathology, Faculty of Medicine, Fukuoka University, Fukuoka, JPN; 7 Department of Oral Dentistry, Fukuoka Dental College, Fukuoka, JPN

**Keywords:** long covid, chronic cough, tnf-α, il-6, chronic epipharyngitis, epipharyngeal abrasive therapy, sars-cov-2 spike rna

## Abstract

A major target of severe acute respiratory syndrome coronavirus 2 (SARS-CoV-2) is the epipharyngeal mucosa. Epipharyngeal abrasive therapy (EAT) is a Japanese treatment for chronic epipharyngitis. EAT is a treatment for chronic epipharyngitis in Japan that involves applying zinc chloride as an anti-inflammatory agent to the epipharyngeal mucosa. Here, we present a case of a 21-year-old man with chronic coughing that persisted for four months after a diagnosis of mild coronavirus disease 2019 (COVID-19), who was treated by EAT. We diagnosed chronic epipharyngitis as the cause of the chronic cough after the SARS-CoV-2 infection. SARS-CoV-2 spike RNA had persisted in the epipharyngeal mucosa of this Long COVID patient. EAT was performed once a week for three months, which eliminated residual SARS-CoV-2 RNA and reduced epipharyngeal inflammation. Moreover, a reduction in the expression of proinflammatory cytokines was found by histopathological examination. We speculate that the virus was excreted with the drainage induced by EAT, which stopped the secretion of proinflammatory cytokines. This case study suggests that EAT is a useful treatment for chronic epipharyngitis involving long COVID.

## Introduction

Some people infected with severe acute respiratory syndrome-coronavirus 2 (SARS-CoV-2) that causes coronavirus disease 2019 (COVID-19) might have symptoms that last a long time afterward, known as Long COVID [1]. Long COVID can be defined as the presence of signs and symptoms even after 12 weeks of SARS-CoV-2 infection and beyond [2]. Cough is a frequent symptom of COVID-19 and can persist for months after infection [3,4]. Vagus nerve stimulation by inflammatory cytokines released from inflammatory cells has been speculated as a cause of cough in COVID-19 [3]. The epipharynx, which is the infection target of SARS-CoV-2 [5,6], has dense vagus nerves under the mucosa [7,8], and chronic inflammation of the epipharynx causes coughing [9].

Epipharyngeal abrasive therapy (EAT) is a treatment for chronic epipharyngitis that has been performed mainly by otorhinolaryngologists in Japan since the 1960s and is effective for upper respiratory symptoms, including chronic cough [9]. Drainage of submucosal inflammatory substances is one of the therapeutic mechanisms by abrasion of the epipharyngeal mucosa with a cotton swab coated with anti-inflammatory zinc chloride [10]. In fact, continuous EAT suppresses submucosal inflammatory cytokines in the epipharynx [11]. Here, we report a case in which continuous EAT was effective for a cough that persisted for four months after SARS-CoV-2 infection, based on immunohistological considerations.

## Case presentation

**Figure 1:**
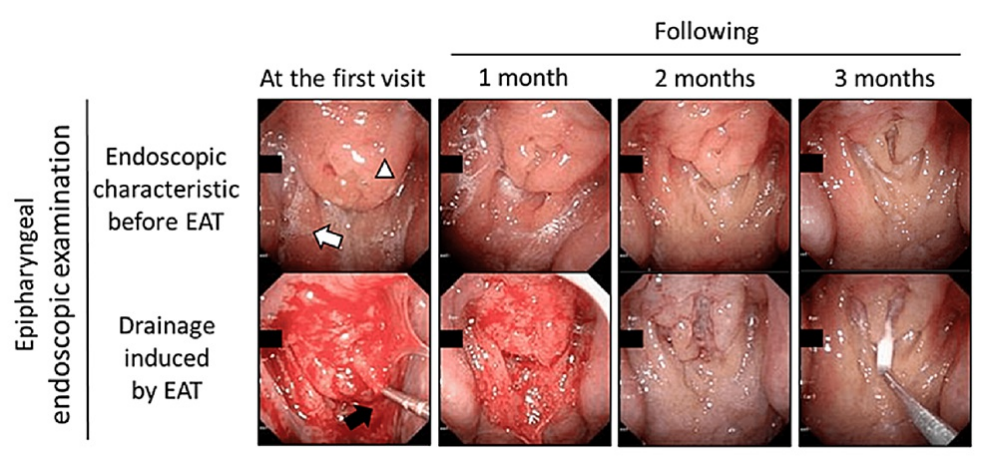
Effect of Epipharyngeal abrasive therapy (EAT) in the patient. Upper panels show endoscopic characteristics before EAT at the first visit and 1, 2, and 3 months. The white triangle indicates swelling of the epipharyngeal mucosa. The white arrow indicates mucus adhesion. Lower panels show bleeding induced by EAT at the first visit and 1, 2, and 3 months. The black arrow indicates a sterile nasal cotton swab containing zinc chloride.

A 21-year-old man presented with chronic coughing that persisted for four months after being diagnosed with mild coronavirus disease 2019 (COVID-19) by an antigen test and clinical symptoms. His past medical history was insignificant. Vital signs, blood tests, chest X-ray, and respiratory function test were normal. A sinus CT scan revealed mucosal thickening of the bilateral maxillary ethmoid and frontal sinus (modified Lund-Mackay score 21/54). Epipharyngeal endoscopic examination showed marked epipharyngeal swelling and a postnasal drip (Figure 1).

**Figure 2:**
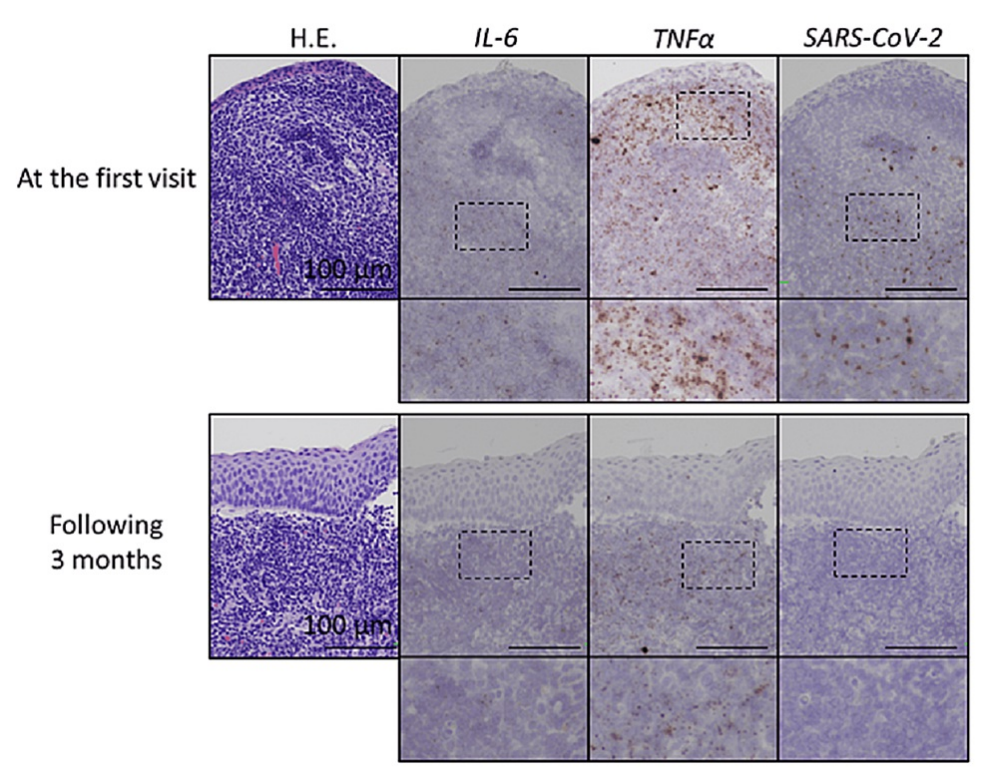
Expression patterns of interleukin 6 (IL-6) and tumor necrosis factor-α (TNF-α), and SARS-CoV-2 mRNAs in the epipharynx in the patient. Expression patterns of IL-6 and TNF-α, and SARS-CoV-2 mRNAs (brown dots) in the epipharynx (Upper panels) at the first visit and (Lower panels) at three months. Magnified images are shown in each lower panel. RNA scope (in situ hybridization system, Advanced Cell Diagnostics, Hayward, CA, USA; IL-6: No. 310371, TNF-α: No. 310421, and SARS-CoV-2: No. 848561) was used following the manufacturer’s guidelines.

Histopathological examination of the epipharyngeal mucosa revealed hyperplastic lymphoid tissue and high expression of interleukin 6 (IL-6) and tumor necrosis factor-α (TNF-α). Additionally, in situ hybridization revealed residual severe acute respiratory syndrome coronavirus 2 (SARS-CoV-2) spike RNA in the epipharynx (Figure 2). Chronic epipharyngitis and chronic sinusitis after SARS-CoV-2 infection were diagnosed as the cause of the chronic cough. Epipharyngeal abrasive therapy, a treatment for chronic epipharyngitis, was performed once a week for three months. Chronic sinusitis was treated with low-dose macrolides, carbocisteine, mometasone furoate, and nasal irrigation. At three months after the start of treatment, histopathological examination showed a reduction in the expression of proinflammatory cytokines. Additionally, SARS-CoV-2 spike RNA persistence was confirmed before treatment had disappeared (Figure 2).

**Figure 3:**
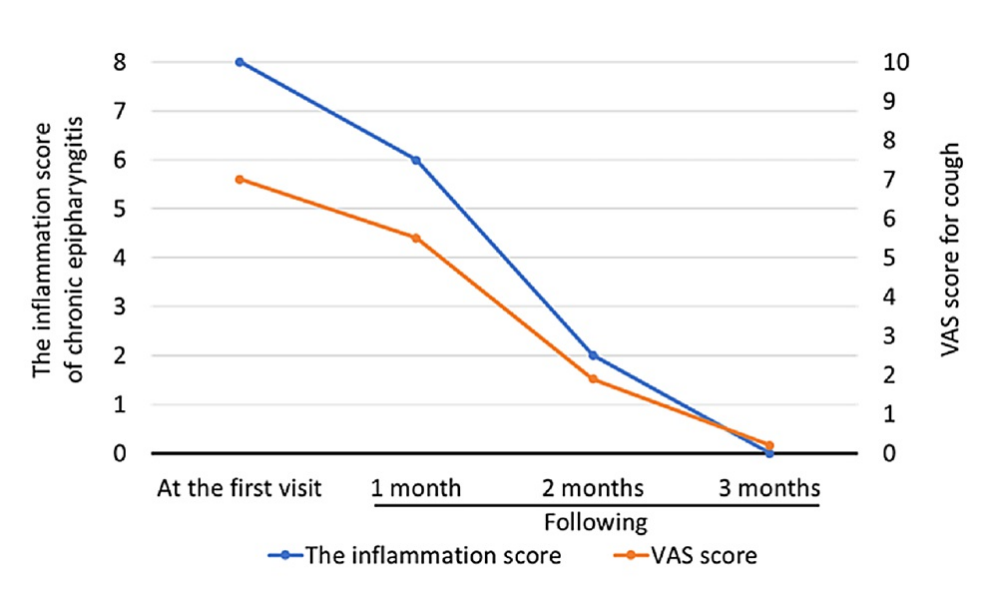
Improvement of epipharyngeal inflammation and cough by Epipharyngeal abrasive therapy (EAT). The inflammation of chronic epipharyngitis was scored by the Japan Society of Stomato-pharyngology EAT Review Committee's assessment criteria for 1) Redness of the epipharyngeal mucosa, 2) swelling of the epipharyngeal mucosa, 3) mucus or crust adhesion, and 4) bleeding during abrasion. Each was rated on a three-point scale (0: none; 1: mild-moderate; 2: severe). The visual analog scale (VAS) for coughing was scored from 0 (absence of symptoms) to 10 (highest severity of symptoms).

His epipharyngeal inflammation had improved (Figure 3) and the chronic sinusitis had also improved (modified Lund-Mackay score: 21/54→3/54). The visual analog scale (VAS) score for coughing improved from 7.0 to 0.2 (Figure 3).

## Discussion

The epipharynx, located behind the nasal cavity, is responsible for mucosal immunity through both innate and acquired immunity as a component of mucosa-associated lymphoid tissue (MALT) [5,11,12]. Meanwhile, the epipharyngeal ciliated epithelium is a major target of severe acute respiratory syndrome coronavirus 2 (SARS-CoV-2) because of its high expression of viral entry factors angiotensin-converting enzyme 2 (ACE2) and transmembrane protease serine 2 (TMPRSS2) [6]. Chronic epipharyngitis is considered a residual immune response after upper respiratory tract infections including coronavirus disease 2019 (COVID-19) and causes various upper respiratory tract symptoms [13]. In fact, it has been reported that >90% of Long COVID patients suffer from moderate-to-severe chronic epipharyngitis [13]. Endoscopic findings such as mucosal redness, swelling, and mucus adhesion are important for the diagnosis of chronic epipharyngitis [13,14].

Epipharyngeal abrasive therapy (EAT) is a Japanese treatment for chronic epipharyngitis, which abrades the epipharyngeal mucosa with a cotton swab containing zinc chloride with anti-inflammatory effects [6,9,11,13-15]. Continuous EAT down-regulates the expression of viruses entry factors via squamous metaplasia and reduces major proinflammatory cytokines such as interleukin 6 (IL-6) and tumor necrosis factor-α (TNF-α) in patients with chronic epipharyngitis [6,11,15]. As a consequence, EAT improves various upper respiratory tract symptoms including a cough associated with chronic inflammation of the epipharynx [9]. Furthermore, it has been reported that the reduction of inflammation of the epipharynx by EAT is also effective for the systemic symptoms of Long COVID [13]. However, the mechanism of residual epipharyngeal inflammation and the efficacy of EAT in Long COVID patients remains unclear [16].

Although it has been reported that SARS-CoV-2 spike RNA persistence in the human body at autopsy [17], this is the first report that SARS-CoV-2 RNA persists in the epipharyngeal mucosa of a Long COVID patient. SARS-CoV-2 antigen persistence in infected tissues affects host immune responses [18] indicating that viral RNA persistence in the epipharynx may be a mechanism for protracted epipharyngitis in Long COVID patients. More interestingly, EAT eliminated residual SARS-CoV-2 RNA and improved epipharyngeal inflammation. We speculate that the viral antigen was excreted along with the drainage induced by EAT and the elimination of the cause of inflammation suppressed the secretion of proinflammatory cytokines, which are closely related to driving cough via neuroimmune interactions [3]. Gabapentin and pregabalin, which are neuromodulators, are considered to be effective in controlling chronic refractory cough in Long COVID [3], whereas we suggest that EAT is a potential novel treatment in terms of removing the cause of cough rather than symptomatic treatment.

As a result, EAT suppressed the inflammation related to persistent viral RNA after infection resolution and the host counter-response, including overproduction of cytokines such as IL-6 and TNF-α, which are presumed to be the cause of Long COVID, in the epipharynx [19]. Chronic cough is a frequent symptom in Long COVID patients and it has been reported that about 10% of their non-hospitalized patients had cough over four months after SARS-CoV-2 infection [20]. Because the epipharynx is anatomically visible only by endoscopic examination, inflammation is often overlooked. In Long COVID patients without lower respiratory tract disorders, who suffer from a chronic cough, it is necessary to focus on upper respiratory tract disorders including chronic epipharyngitis. We propose that EAT is useful as a treatment for chronic epipharyngitis involving Long COVID.

## Conclusions

Epipharynx is a major target of SARS-CoV-2. Severe inflammation and SARS-CoV-2 spike RNA persisted in the epipharynx of a Long COVID patient. Epipharyngeal abrasive therapy (EAT), a treatment for chronic epipharyngitis, was effective for a chronic cough involving Long COVID. EAT suppresses the expression of proinflammatory cytokines in the epipharynx that cause coughing. Surprisingly, EAT cleared residual SARS-CoV-2 RNA in the epipharynx. As a result, EAT may be effective in improving upper respiratory tract symptoms in Long COVID patients.
